# Graphic analysis of various sulfur applications on safflower fatty acids profile

**DOI:** 10.5114/bta.2024.135640

**Published:** 2024-03-29

**Authors:** Naser Sabaghnia, Mostafa Fattahi, Mohsen Janmohammadi, Amin Abbasi

**Affiliations:** Department of Agronomy and Plant Breeding, Faculty of Agriculture, University of Maragheh, Maragheh, Iran

**Keywords:** fatty acids profile, oil percent, treatment by trait biplot analysis

## Abstract

In this study, we examined the effects of seven different sulfur treatments on safflower seeds. The treatments included: no sulfur application (S0), 25 kg/ha of pure bulk sulfur (S25), 50 kg/ha of pure bulk sulfur (S50), 25 kg/ha of sulfur phosphate (Sp25), 50 kg/ha of sulfur phosphate (Sp50), 25 kg/ha of zinc sulfate (Zs25), and 50 kg/ha of zinc sulfate (Zs50). Our evaluation covered various seed quality attributes, including ash percentage (ASH), oil percentage (OIL), and protein percentage (PRO). Additionally, we analyzed the fatty acid composition, including palmitic acid 16 : 0 (PAL), stearic acid 18 : 0 (STE), oleic acid 18 : 1 (OLE), linoleic acid 18 : 2 (LINL), arachidic acid 20 : 0 (ARA), and linolenic acid 18 : 3 (LINN). The vector-view of the biplot illustrated positive associations among the fatty acids STE, PAL, and OLE, whereas ASH exhibited negative associations with OIL, LINL, and LINN. The polygon-view graph was divided into four sectors, with the genotype S50 emerging as the top performer for attributes such as OIL, PRO, LINL, ARA, and LINN. Treatment Zs50 occupied the vertex of another sector and displayed the highest values for palmitic acid PAL, STE, and OLE, while treatment S0 was positioned at the vertex of the next sector, characterized by its high ASH content. By utilizing the ideal tester tool of treatment by trait biplot, we identified OIL as the desirable trait that most effectively represented the data. The qualitative properties of safflower oil were notably influenced by sulfur application, with treatment S50 proving to be the most effective in enhancing these properties.

## Introduction

Safflower (*Carthamus tinctorius* L.) stands as a significant oilseed crop with diverse applications, including the production of edible oil and medicinal uses. Investigating the morphological variations within safflower populations is imperative to enhance breeding strategies, preserve genetic diversity, and develop superior cultivars (Ebrahimi et al., 2023). Interestingly, a comparison of safflower cultivation areas and seed production worldwide reveals a decline in the cultivated area by approximately 350 000 ha and a reduction in safflower seed production by about 300 000 tons over the past 5 years (FAOSTAT, 2022). Safflower seeds exhibit significant morphological diversity, including variations in size, shape, color, and surface texture, all of which can impact seed quality, yield performance, and suitability for various end uses (Baljani et al., 2016). The exploration of this morphological variation enables researchers to identify genetic markers associated with desirable traits, facilitating more efficient breeding practices and the development of improved cultivars. Moreover, understanding the diversity in morphological and fatty acid profiles within safflower populations is vital for germplasm conservation, ensuring the preservation of unique genetic resources that may prove valuable for future crop enhancement efforts (Hassani et al., 2020).

Low soil fertility and inadequate soil health conditions can significantly jeopardize food security and lead to nutritional imbalances (Janmohammadi and Sabaghnia, 2023). Addressing nutrient deficiency in semiarid areas becomes imperative, requiring a top priority in agricultural and soil management programs. Semiarid soils are characterized by a very slow rate of soil formation, low soil organic matter content, limited waterholding capacity, and a reduced ability to supply nutrients (Ayangbenro and Babalola, 2021). Sulfur plays a crucial role in the biosynthesis of various secondary metabolites, sulfatides, and numerous vitamins. Despite its essential role, sulfur is often considered as a minor nutrient in crop development and is frequently overlooked by local farmers (Li et al., 2020).

Sulfur serves several functions in plants. It actively participates in amino acid and protein synthesis, assumes a fundamental role in chlorophyll biosynthesis, proves indispensable in oil synthesis within oilseed crops, and has interconnected roles in nitrogen and carbon metabolism (Nakajima et al., 2019). Notably, oilseeds, compared to cereals, have a higher demand for sulfur, primarily due to the extensive biosynthesis of oil, and the appropriate application of sulfur from appropriate sources emerges as an effective crop management strategy, particularly for enhancing safflower yields in semiarid regions (Narayan et al., 2022).

The application of sulfur brings about improvements in the release of other elements from the soil into the rhizosphere, thereby enhancing the absorption rate by plant roots. This effect is attributed to sulfur’s influence on soil acidity and other chemical properties. However, there is limited information available regarding the different levels and types of sulfur fertilizer application in safflower cultivation, particularly in semiarid conditions. This investigation was performed to gain a better grasp of the positive sulfur impacts of its application at various levels and from various sources – such as sulfur phosphate, zinc sulfate, and pure bulk sulfur – on the oil percentage of seeds and the fatty acid profiles extracted from safflower seeds.

## Materials and methods

### Trial protocol

The trial was conducted following a randomized complete block design with three replications, involving ten random samples from each plot in the field located in Baneh, Iran (35˚59′N, 45˚53′E, altitude: 1610 m). The region experienced a total rainfall of 150 mm during the growing season. Sulfur treatments included no sulfur application as a control (S0), 25 kg/ha of pure bulk sulfur (S25), 50 kg/ha of pure bulk sulfur (S50), 25 kg/ha of sulfur phosphate (Sp25), 50 kg/ha of sulfur phosphate (Sp50), 25 kg/ha of zinc sulfate (Zs25), and 50 kg/ha of zinc sulfate (Zs50).

The experimental field was plowed using a moldboard plow in the autumn season, and 20 t/ha of farmyard manure was applied. Subsequently, the designated sulfur treatments were applied before planting. Safflower seeds (Chinese cultivar ZY-S) were subjected to disinfection with benomyl fungicide and manually sown at the end of April 2021, followed by irrigation. Row spacing was set at 50 cm, with 10 cm within-row spacing, and manual weed control was carried out as required throughout the crop’s development. Irrigation was carried out at intervals of every 4–10 days up until the flowering stage.

### Biochemical analysis of oil

For chemical and physical analysis, oil extraction was conducted using the cold extraction method with industrial hexane. In the initial stage, samples were combined with hexane at a ratio of 1 : 5 (oil : hexane) and stirred on a magnetic stirrer at a medium speed for 24 h. To quantify different fatty acids in the samples, gas chromatography methods were used. The preparation of methyl ester of fatty acids was done according to the method reported by (Ortega et al., 2004). Initially, 50 ml of the oil sample was poured into a lidded test tube, and 1 ml of hexane was added. After the complete dissolution of the oil, 100 μl of methanolic sodium methoxide was added, and the mixture was shaken for 15 min at room temperature. Following the required time for the appearance of two distinct layers inside the tube, the hexane phase was taken out and transferred to another test tube containing sodium sulfate to eliminate excess moisture. During injection into the device, 1 μl of the hexane phase was used.

For determining the fatty acid profile in oil samples, a gas chromatography device (Agilent 6890N, USA) equipped with an FFAP-TC capillary column (30 m in length, 0.32 mm in diameter, and a thickness of the thin layer inside the tube (phase constant) of 0.25 μm) was used. The detector, an FID type device, operated at 250˚C, and nitrogen served as the carrier gas. The thermal program used was as follows: starting the program with a temperature of 150˚C and staying at the same temperature for 1 min, then increasing the temperature to 190˚C at a rate of 5˚C/min, and a 2-min hold at this temperature. Subsequently, the temperature was increased again at a rate of 5˚C/min up to 250˚C, and it remained at this temperature for 8 min. The methylated sample was injected into the device as a single unit, with a volume of 11 μl. The mean values of the measured parameters are given in Table 1.

**Table 1:** Mean of safflower oil characteristics grown under sulfur treatments

Treatments	Ash [%]	Oil [%]	Pro [%]	Pal [%]	Ste [%]	Ole [%]	Linl [%]	Ara [%]	Linn [%]
S0	3.70	24.60	21.02	5.55	1.39	7.76	78.41	0.18	0.12
SP25	3.31	25.16	21.05	5.74	1.44	8.32	78.99	0.20	0.14
SP50	3.13	25.36	22.11	6.05	1.51	8.32	79.92	0.22	0.14
S25	3.25	26.14	22.40	6.45	1.73	9.00	79.01	0.22	0.14
S50	2.82	26.43	22.81	6.26	1.73	8.72	81.19	0.90	0.16
ZS25	2.77	25.17	22.26	6.01	1.87	8.33	80.27	0.23	0.14
ZS50	2.82	25.65	22.52	6.29	2.01	8.76	80.75	0.24	0.15

ASH – ash percent, OIL – oil percent, PRO – protein percent, PAL – palmitic acid 16 : 0, STE – stearic acid 18 : 0, OLE – oleic acid 18 : 1, LINL – linoleic acid 18 : 2, ARA – arachidic acid 20 : 0, LINN – linonenic acid 18 : 3

### TT biplot

The obtained data on the fatty acid profile were subjected to a treatment by trait (TT) interaction biplot analysis using the GGEbiplot software (Yan, 2001), according to the following formula:


$$\frac{X_{ij}-\mu_{j}}{S_{j}}=\sum_{n=1}^{2}\alpha_{n}\beta_{in}\gamma_{jn}+E_{ij}$$

In the formula provided: *X_ij_* represents the mean of treatment *i* for trait *j*, *μ_j_* is the mean of all treatments for trait *j*, *S_j_* is the standard deviation of trait *j* among treatments, *α_n_* is the singular value for PC *n*, *β_in_* and *η_jn_* are scored for treatment *i* and trait *j* on PC *n*, respectively, *E_ij_* is the residual magnitude of the model related to treatment *i* for trait *j*. To achieve symmetric scaling in the values of both treatments and traits, the singular value *α_n_* needs to be adjusted via absorption of their vectors. The TT interaction biplot graphs are created by plotting the symmetrically scaled scores of the genotypes and traits. In these graphs, each treatment (entry) or trait (tester) is represented by a unique marker, allowing for a graphical indication of the associations between traits as well as treatments. For a more overall grasp of the TT biplot, you can refer to Yan and Frégeau-Reid (2018) for additional information.

## Results and discussion

The TT biplot model revealed that the first two PCs accounted for 72 and 12% of the observed variation, summing up to 84% in the interaction of treatments and traits. When ANOVA indicates significant differences in interactions, the TT biplot method is advisable; however, if the interaction is not significant, the TT biplot procedure may not be recommended. The ANOVA analysis for safflower (Table not shown) confirmed the appropriateness of using the TT biplot, as the traits showed significance among treatments.

**Fig. 1:**
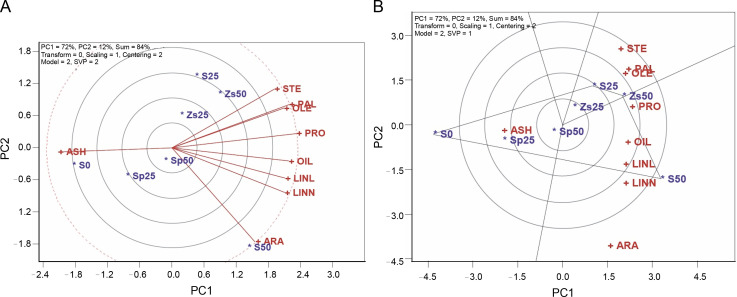
A) relations among testers and B) polygon-view of TT biplot for safflower traits

The relationship-among-testers tool in the TT biplot model (Fig. 1A) represents the relationships among safflower traits. In this tool, the traits’ rays connected to the plot center represent the traits’ vectors, and their relationships are assessed by the magnitude of the angle cosine between vectors. Traits or testers are considered related positively when the internal angle between markers is less than 90˚, related negatively when the angle exceeds 90 ˚, and are not related to the angle of 90 ˚. Longer vectors indicate more significance and responsiveness, while shorter vectors are less significant and responsive. Traits located at the plot center have no significant relationship with other traits.

Figure 1A shows that stearic acid 18 : 0 (STE), palmitic acid 16 : 0 (PAL), and oleic acid 18 : 1 (OLE) traits are positively related, as indicated by the small acute angle markers. Additionally, oil percent (OIL), linoleic acid 18 : 2 (LINL), and linolenic acid 18:3 (LINN) show positive correlations with tiny acute angles of markers. Conversely, there is a negative association between ash percent (ASH) and OIL, LINL, and LINN, as well as between protein percent (PRO) and ASH, as evidenced by the relatively large obtuse angles of markers (Fig. 1A).

The TT biplot model’s predictions for trait associations align well with those reported in Pearson’s correlation matrix (Table 2). However, there are some minor inconsistencies, attributed to the fact that the model accounts for 84% of the observed variability rather than 100%. It is worth noting that safflower oil primarily consists of palmitic acid, stearic acid, and oleic acid, which are positively associated, making safflower oil a favorable source of healthy fats that can positively impact cholesterol levels (Amirkhiz et al., 2021). Additionally, the stability of such fatty acids was better than others, suggesting high frictional properties for the long maintenance of produced oil.

**Table 2:** Correlation coefficients among measured traits of safflower

	Ash	Oil	Pro	Pal	Ste	Ole	Linl	Ara
Oil	−0.561*							
Pro	−0.821	0.820						
Pal	−0.649	0.880	0.907					
Ste	−0.828	0.506	0.792	0.736				
Ole	−0.582	0.897	0.775	0.949	0.669			
Linl	−0.927	0.594	0.819	0.583	0.712	0.487		
Ara	−0.453	0.692	0.552	0.354	0.197	0.336	0.660	
Linn	−0.741	0.809	0.695	0.654	0.442	0.702	0.799	0.695

* Critical correlation values, degrees of freedom = 5 and *P* < 0.01 are 0.75 and 0.88, respectively; ASH – ash percent, OIL – oil percent, PRO – protein percent, PAL – palmitic acid 16 : 0, STE – stearic acid 18 : 0, OLE – oleic acid 18 : 1, LINL – linoleic acid 18 : 2, ARA – arachidic acid 20 : 0, LINN – linonenic acid 18 : 3

The polygon tool within the TT biplot aids in exploring the best treatments for one or more traits and facilitates a visual understanding of the interaction between traits and treatments. In Figure 1B, the polygon is divided into four sectors, and the sector corresponding to S50 (50 kg/ha of pure bulk sulfur) emerges as the superior performer for oil percent (OIL, 26.4%), protein percent (PRO, 22.8%), linoleic acid 18 : 2 (LINL, 81.2%), arachidic acid 20 : 0 (ARA, 0.89%), and linolenic acid 18 : 3 (LINN, 0.16%). Treatment Zs50 (50 kg/ha zinc sulfate) is positioned on the vertex of another sector, displaying the highest values for traits such as palmitic acid 16 : 0 (PAL, 6.3%), stearic acid 18 : 0 (STE, 2.0%), and oleic acid 18 : 1 (OLE, 8.8%), while treatment S0 (no sulfur application) is situated on the vertex of a different sector concerning ash percent (ASH, 3.7%) (Fig. 1B).

The treatments identified as vertices in each section represent the highest-performing treatments for the traits within that section. In contrast, other vertex genotypes did not excel in any of the traits and performed poorly in some or all of them. Treatment S25 (25 kg/ha of pure bulk sulfur) did not excel in any traits (Fig. 1B). Considering the significance of OIL (26.1%), LINL (79.0%), and LINN (0.14%) in safflower’s quality and quantity performance, it appears that using 50 kg/ha of pure bulk sulfur is advisable for safflower production in semiarid regions. Sulfur significantly affects the metabolism of linoleic acid enzymes and bond formation reactions for oil production, and its application has been demonstrated to increase beneficial fatty acids as well as oil percentage (Chaudhary et al., 2023).

**Fig. 2:**
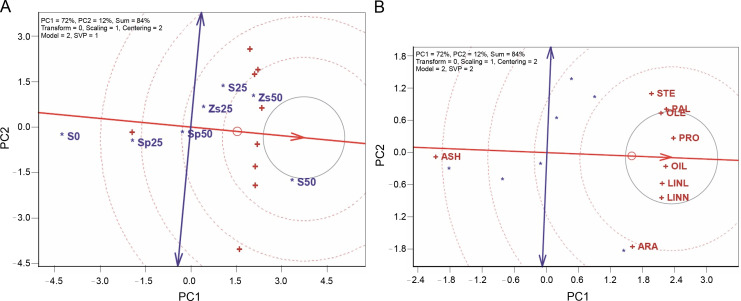
A) ideal treatment and B) ideal trait of TT biplot for safflower under sulfur treatments

Figure 2A shows the ideal entry in the TT biplot, represented by a projection on the axis (the largest entry vector), indicating above-average performance with a low projection on the vertical axis. The best treatment, located nearby, is treatment S50 (50 kg/ha of pure bulk sulfur), followed by treatments Zs50 (50 kg/ha zinc sulfate), S25 (25 kg/ha of pure bulk sulfur), and Zs25 (25 kg/ha zinc sulfate), exhibiting higher quality than other treatments. Conversely, lower-grade treatments were S0 (no sulfur application), Sp25 (25 kg/ha of sulfur phosphate), and Sp50 (50 kg/ha of sulfur phosphate), positioned farther from the ideal entry (Fig. 2A). The desirability of treatment S50 is reinforced by both the polygon-view and ideal-entry-view of the TT biplot model. Traits such as OIL, LINL, and LINN, crucial for safflower quality and quantity, were significantly higher when applying 50 kg/ha of pure bulk sulfur. This increase is likely due to sulfur’s essential role in amino acid biosynthesis, influencing oil percentage. Sulfur application in its elemental form may prevent floral abortion, leading to a higher number of filled seeds and a more robust establishment (Nawaz et al., 2020). Sulfur is a component of some amino acids, essential for protein synthesis, and its shortage can influence crop performance.

In Figure 2B, an ideal tester, representing the ideal trait, should ideally be positioned on the mean traits coordinate axis. A trait is considered preferable if it is located near the ideal tester. Consequently, oil percent (OIL) is a desirable trait, followed by protein percent (PRO), linoleic acid 18 : 2 (LINL), and linolenic acid 18 :3 (LINN). Oleic acid 18 : 1 (OLE) and palmitic acid 16 : 0 (PAL) are relatively desirable traits, while stearic acid 108 (STE), arachidic acid 20 : 0 (ARA), and ash percent (ASH) are relatively undesirable traits (Fig. 2B). These results align with the findings of Baljani et al. (2016), identifying oil percent as the ideal trait in the study of 64 safflower genetic variations. Consequently, OIL should be a focal point in safflower breeding programs and for determining selection indices. It can also be practically used for evaluating safflower responses to various treatments whereas evaluation of oil percent alone can detect differences among treatments.

Our findings demonstrate that the application of sulfur fertilizer leads to a reduction in ash content in safflower achenes, while simultaneously increasing protein and oil contents. This observation suggests that effective management of essential nutrients can influence the distribution of photoassimilates, ultimately enhancing economic performance. Fertilizer management, especially in oilseeds, is crucial, given our research indicating that the response of oil quality to sulfur from different sources can vary (Janmohammadi et al., 2017). To achieve optimal performance and the desired quality, it is essential to develop a balanced nutrition plan for safflower. In this study, the highest quality was observed when applying high levels of pure bulk sulfur, aligning with the report of Ullha et al. (2022), who reported the highest yields with high levels of sulfur fertilizer.

Notably, our study involved the application of farmyard manure, and it seems that sulfur fertilizer’s efficiency is enhanced under such conditions due to increased availability in the rhizosphere environment. In these conditions, farmyard manure may contribute to boosting sulfur absorption and improving its utilization efficiency by adjusting chemical conditions (Ashraf et al., 2016). The results indicate that optimizing the supply of one element enhances the efficiency of another limiting element due to synergistic relationships among elements. This adjustment of source – sink relationships lead to improved oil quality, as suggested by Grzebisz et al. (2022).

The observed increase in oil content in safflower with higher levels of sulfur, particularly at higher sulfur levels (45 kg/ha), aligns with the findings of Nathan et al. (2017). The increase in oil content with higher sulfur levels can be attributed to higher seed yield. The application of sulfur to crops is known to enhance the formation of acetyl coenzyme A, a precursor compound crucial for the synthesis of long-chain fatty acids, resulting in increased oil percentage (Panda et al., 2000).

## Conclusion

The qualitative properties of safflower oil exhibited positive responses to the sulfur treatments, with the application of 50 kg/ha of pure bulk sulfur (S50) emerging as the most effective treatment. The utilization of the TT biplot model proved to be an excellent graphical tool for analyzing the interaction between traits and treatments. This approach facilitated a comprehensive understanding of the structure of the two-way layout traits and their responses to varying sulfur treatments.

## Conflict of interest

The authors declare no conflict of interest.
